# Hematological and Clinical Features Associated with Initial Poor Treatment Outcomes in Visceral Leishmaniasis Patients with and without HIV Coinfection in Gondar, Northwest Ethiopia

**DOI:** 10.3390/tropicalmed8010036

**Published:** 2023-01-04

**Authors:** Muluneh Ademe, Yaneth Osorio, Rawliegh Howe, Saba Atnafu, Tadele Mulaw, Helina Fikre, Bruno L. Travi, Asrat Hailu, Peter C. Melby, Tamrat Abebe

**Affiliations:** 1 Department of Microbiology, Immunology & Parasitology, College of Health Sciences, Addis Ababa University, Addis Ababa P.O. Box 9086, Ethiopia; 2 Division of Infectious Diseases, Department of Internal Medicine, University of Texas Medical Branch, 301 University Boulevard, Route 0435, Galveston, TX 77555, USA; 3 Aramuer Hanson Research Institute (AHRI), Addis Ababa P.O. Box 1005, Ethiopia; 4 Leishmaniasis Research and Treatment Center, University of Gondar, Gondar P.O. Box 196, Ethiopia

**Keywords:** visceral leishmaniasis, HIV coinfection, sepsis, dyspnea, poor outcomes, AmBisome, miltefosine

## Abstract

Ethiopia is among the countries with a high leishmaniasis burden. In this retrospective review, we aimed to determine hematological and clinical features associated with initial poor treatment outcomes of visceral leishmaniasis (VL) patients. The majority of VL cases in this study had leucopenia (94.3%), thrombocytopenia (87.1%), and anemia (85.9%). HIV coinfection was present in 7.0% (*n* = 23) of VL cases. At the center, VL patients without HIV coinfection were treated with sodium stibogluconate and paromomycin combination, whereas HIV coinfected cases were treated with AmBisome and miltefosine combination therapy. End-of-treatment cure rates among HIV-positive and HIV-negative visceral leishmaniasis cases, respectively, were 52.2% and 96.9%. Case fatality rates were 34.8% and 2.7% in HIV-positive and HIV-negative cases, respectively. Overall, non-survivors in this study were more likely to have HIV (55.0% vs. 4.1%, *p* < 0.001), sepsis (15.0% vs. 1.4%, *p* = 0.019), and dyspnea (40.0% vs. 2.7%, *p* < 0.001) at admission. In this regard, particular attention to the management of superimposed disease conditions at admission, including sepsis, HIV, and dyspnea, is needed to improve VL patients’ treatment outcomes. The inadequacy of the current treatments, i.e., AmBisome and miltefosine combination therapy, for HIV coinfected visceral leishmaniasis patients requires further attention as it calls for new treatment modalities.

## 1. Introduction

Visceral leishmaniasis (VL) is a protozoan infection characterized by prolonged fever, anemia, weight loss, and hepatosplenomegaly. VL is a neglected tropical disease that mainly affects the impoverished population globally. VL is the most severe form of leishmaniasis, and is invariably fatal if left untreated [1,2,3]. The majority of new VL cases (90%) reported to WHO by the year 2020 occurred in India, Brazil, Ethiopia, South Sudan, Kenya, Sudan, Eritrea, Somalia, China, and Yemen [3].

*Leishmania donovani* is regarded as the cause of VL in many endemic regions of Ethiopia [4]. The transmission and burden of VL in Ethiopia vary geographically, which is mostly associated with the socio-economic status, immune status, nutritional status, migration of seasonal workers to and from endemic areas, and the presence of vectors [5,6,7]. The annual incidence of VL in Ethiopia is estimated to exceed 4000 cases, and northwest Ethiopia, the site of this study, contributes to the highest number of cases in the country.

VL has a complex pathophysiology, and the clinical manifestations range from asymptomatic or subclinical infections to serious and fatal diseases, which are determined by the different host and parasitic factors [8,9,10,11]. VL is considered an opportunistic parasitic infection of HIV-positive patients. The HIV pandemic is considered one of the primary drivers of the increased risk of developing active VL [12]. Ethiopia, Brazil, and India recorded high *Leishmania*–HIV coinfection rates [3]. The prevalence of HIV coinfection among VL patients in Ethiopia has remained high (20.88% to 24.86% in northwest Ethiopia, as reviewed in [13]) despite the projected decreasing trends in the burden of HIV in the general population [14]. *Leishmania*–HIV coinfection increases the risk of developing full-blown VL, relapse, treatment failures, and mortality [15,16]. In Ethiopia, a high treatment failure rate (30%) was reported in VL–HIV coinfected patients [17]. Regardless of the drug used, most HIV coinfected cases relapse and eventually die [18,19,20]. Anti-leishmanial drugs also showed high toxicity in HIV coinfected VL cases [17,21]. Overall, *Leishmania*–HIV coinfection poses challenges to the VL control effort [3,22,23]. Despite efforts made to identify a new combination of treatments to control the disease, VL with and without HIV coinfection continues to pose a challenge. This retrospective study mainly focused on determining the hematological and clinical features associated with initial poor end-of-treatment outcomes of VL patients with and without HIV coinfection.

## 2. Materials and Methods

### 2.1. Study Design and Setting

This study was conducted at the Leishmaniasis Research and Treatment Center (LRTC) of the University of Gondar, which is located in Gondar town, Amhara regional state of Ethiopia. The University of Gondar LRTC is a major VL diagnosis and treatment center in Ethiopia. A retrospective study was conducted among parasitologically confirmed VL patients diagnosed and treated in LRTC at the University of Gondar from 2019 to 2021. VL patients with and without HIV coinfection who had either primary VL or relapse were included in this study.

### 2.2. Inclusion and Exclusion Criteria

Patients were excluded from the study if (1) parasitologic confirmation was not provided, and if patients were treated because they had a positive serological (rk39) test, (2) patients fulfilled the case definition of VL and were treated with anti-leishmanial drug(s) despite a negative parasitological test result, (3) patients whose HIV status was not known even when they had parasitologically confirmed VL, and (4) patients who were transferred out or defaulted, and thus their clinical outcome could not be determined.

### 2.3. Data Collection and Analysis

A clinical examination and surveillance form was used to collect data from study subjects, including demographic characteristics (age, gender, residence, ethnicity, marital status), data on clinical presentations, baseline hematological results, underlying medical conditions, type and duration of treatment provided, and treatment outcomes. Data were entered and analyzed with SPSS version 21 and GraphPad prism version 9. Chi-square and Wilcoxon tests or unpaired Student’s t-tests were used to analyze categorical and continuous variables, respectively. Bivariate analysis was performed to analyze predictors of poor clinical outcomes, with the results expressed as crude odds ratios. Then, variables with significant associations in the bivariate analysis were further subjected to multivariate analysis, with the results expressed as adjusted odds ratios. Variables with *p*-values < 0.05 were considered independent predictors of poor end-of-treatment outcomes.

### 2.4. Definitions and Reference (Normal) Values

Case definition of VL: A patient who either lives or has traveled to a VL endemic area and presents with fever for more than 2 weeks; has splenomegaly and/or lymphadenopathy, and/or loss of appetite, and/or weight loss, and/or anemia, and/or leucopenia, and/or thrombocytopenia. VL patients in our case are those who fulfill the case definition of VL and who had a positive parasitological test (which is conducted by microscopic demonstration of amastigotes in Giemsa-stained tissue aspirates of the spleen, bone marrow, or lymph node). Parasite grade (PG) 6+ = >100 parasites per field, 5+ = 10–100 parasites per field, 4+ = 1–10 parasites per field, 3+ = 1–10 parasites per 10 field, 2+ = 1–10 parasites per 100 field, 1+ = 1–10 parasites per 1000 field, negative = 0 parasite per 1000 field. VL and HIV coinfection refers to patients who had laboratory-confirmed VL and HIV infections during the same hospitalization period. Treatment outcomes were grouped as (1) good if a patient is clinically cured at the end of treatment, (2) poor for a VL patient with treatment failure and/or death. Clinical cure was assessed at the end of treatment, and refers to the absence of fever and hematological abnormalities, and a significant regression in the size of the enlarged spleen and liver. “Clinical Cure” used here or the term “cure” as used in the main body of the manuscript can be taken as synonymous with “initial cure”. Treatment failure refers to VL patients who are unresponsive to antileishmanial treatment. Mixed cell refers to a combined value of monocytes, basophils, and eosinophils. Unless indicated, reference (normal) intervals used by the LRTC were included for the study analysis, including WBC (count/µL) 3200–8800, Hgb (g/dL) 11–18, platelet (count/µL) 128,000–432,000, lymphocyte (count/µL) 1000–3700, neutrophil (count/µL) 1500–7000, monocyte (count/µL) 0–700, mixed cell (count/µL) 0–1200, aspartate transaminase (AST, IU/L) 0–37, alanine transaminase (ALT, IU/L) 0–42, blood urea nitrogen (BUN, mg/dL) 10–50, prothrombin time (PT, in sec) 10–14, creatinine (mg/dL) 0.6–1.1, K+ (mEq/L) 3.5–5.2, Na+ (mEq/L) 135–145. Malnutrition was calculated using body mass index (BMI, kg/m^2^) values as in [24,25]. Malnutrition, BMI ≤ 18.5; severe malnutrition, BMI < 15.5.

## 3. Results

### 3.1. Demographic Characteristics

**Figure 1:**
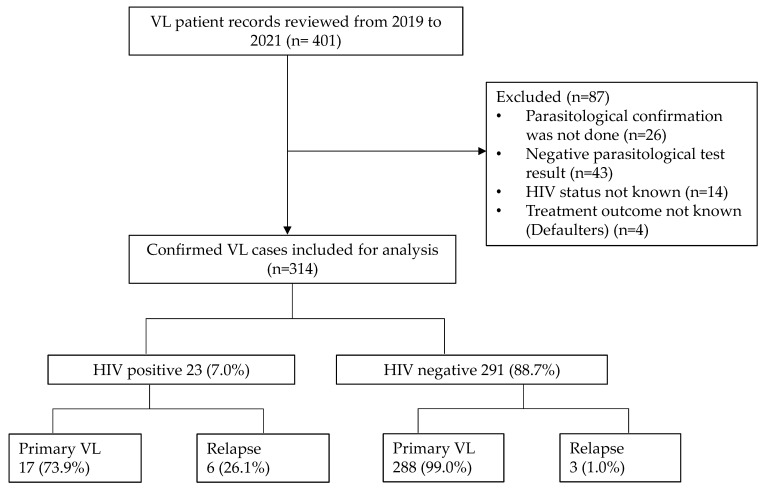
Data collection flowchart of VL patients (LRTC, Gondar, Ethiopia).

We reviewed the records of a total of 401 patients who had been diagnosed and treated for VL in the LRTC from 2019 to 2021. Among these, 87 cases were excluded from the analysis due to the fact that (i) 26 VL patients had a positive serological (rk39) test but parasitological confirmation was not provided, (ii) 43 patients had a negative parasitological test result, (iii) HIV status was not known in 14 VL patients, and (iv) 4 VL patients were defaulters (Figure 1). Thus, a total of 314 parasitologically confirmed VL cases were included in the study.

The majority of VL patients were males (*n* = 311, (99.0%)) and rural residents (*n* = 298, (94.9%)). The ages of VL patients ranged from 14 to 65 years, with a median (interquartile range (IQR)) age of 23 (7) years. Twenty-three (7.0%) VL patients were HIV-positive. At admission, 19 (82.6%) of HIV cases were on antiretroviral therapy (ART). CD4 count was available for eight (34.78%) HIV-positive VL patients, of which seven (87.5%) had CD4 counts <200 cells/μL. About a quarter (*n* = 6, (26.1%)) of HIV coinfected VL cases and 1.0% (*n* = 3) HIV-negative VL cases had relapsed VL. Diagnosis of VL was confirmed by a parasitological examination of splenic aspirate in 263 (83.8%), bone marrow aspirate in 49 (15.6%), and lymph node aspirate in 2 (0.6%) patients. The median (IQR) splenic parasite grade (PG) of HIV coinfected VL cases and HIV-negative VL cases was 5 (3) and 2 (2), respectively (Table 1).

### 3.2. Baseline Laboratory Characteristics

**Figure 2:**
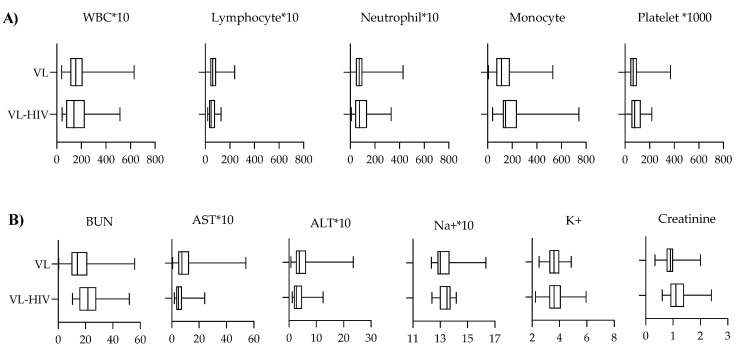
(**A**) White blood cell (WBC) and platelet count, (**B**) clinical chemistry test results among VL patients with and without HIV coinfection. * 10, the presented cell count value should be multiplied by 10 to obtain the actual cell count.

**Figure 3:**
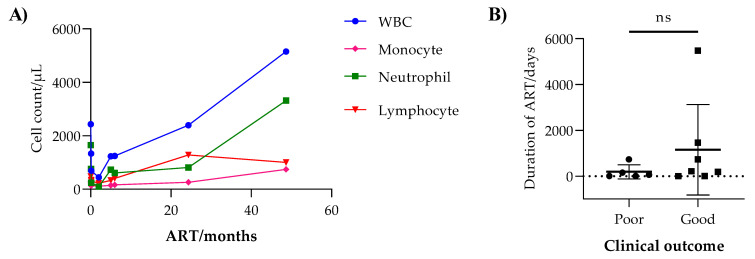
(**A**) Correlation of ART duration with total leucocyte count (r = 0.9, *p* = 0.003), monocyte count (r = 0.9, *p* = 0.0023), neutrophil count (r = 0.8, *p* = 0.017), and lymphocyte count (r = 0.8, *p* = 0.0155) among HIV-positive VL patients, (**B**) end-of-treatment clinical outcomes with respect to ART duration. ns, not significant.

The majority of VL cases in this study had hematological abnormalities. Leucopenia (absolute WBC counts < 3200/µL) was present in 94.3%, thrombocytopenia (platelet count < 128,000/µL) in 87.1%, and anemia (Hgb < 11 mg/dL) in 85.9%. Both HIV-positive and HIV-negative VL patients had leucopenia (87.0% and 94.8%, respectively), neutropenia (80.0% and 91.8%, respectively), and lymphocytopenia (95.2% and 82.4%, respectively) (Table 1 and Figure 2A). We observed a significant increase in the leucocyte counts of HIV-positive VL patients through an increase in the duration of ART (Figure 3A). In both HIV-positive and HIV-negative VL patients, the mean AST was significantly higher (63.3 ± 52.8 IU/L and 109.2 ± 104.7 IU/L, respectively, *p* ≤ 0.026) than the AST reference value (37 IU/L) (Figure 2B and Table 2).

### 3.3. Clinical Presentations of VL Patients with and without HIV Coinfection

Most VL patients in this study (96.8%) presented with fever lasting more than 2 weeks. Nearly all HIV-negative VL patients (98.3%) and about three quarters (78.3%) of VL–HIV coinfected patients presented with fever lasting more than 2 weeks. A high proportion of HIV-positive and HIV-negative VL patients had splenomegaly (100.0% and 97.6%, respectively), malnutrition (95.7% and 84.5%, respectively), and cough (69.6% and 57.0%, respectively). Pneumonia and intestinal parasite infections were the most frequently identified superimposed medical conditions at the time of admission among VL cases with and without HIV coinfection (Table 2).

### 3.4. Treatments and Predictors of Poor Treatment Outcomes among VL Patients with and without HIV Coinfection

Most HIV coinfected VL patients (*n* = 22, (95.7%)) were treated with a combination therapy of liposomal amphotericin B (AmBisome) infusion (six doses of 5 mg/kg/day) and oral miltefosine (≥45 kg, 150 mg/day for 28 days; <45 kg, 100 mg/day for 28 days). Eight VL–HIV coinfected cases had either died (*n* = 5) or experienced treatment failure (*n* = 3) before they completed the 28 days AmBisome and miltefosine combination therapy. Fourteen VL–HIV coinfected cases completed the 28 days AmBisome and miltefosine combination therapy (two died, eight were cured, and four cases who did not fully recover during the first 28 days combination therapy received an additional 28 days of AmBisome and miltefosine combination therapy (all cured)). Furthermore, one HIV coinfected patient who was on sodium stibogluconate (SSG) (20 mg/kg/day) and miltefosine therapy also died on the 6th day of therapy.

**Figure 4:**
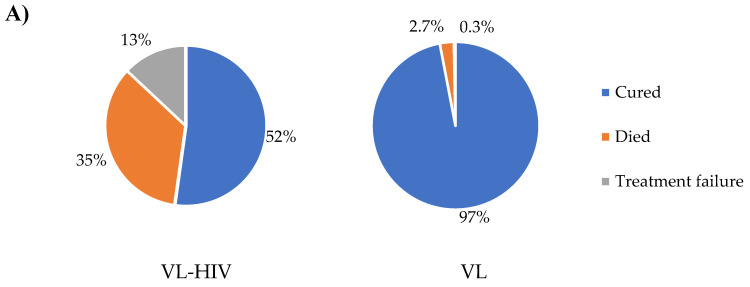

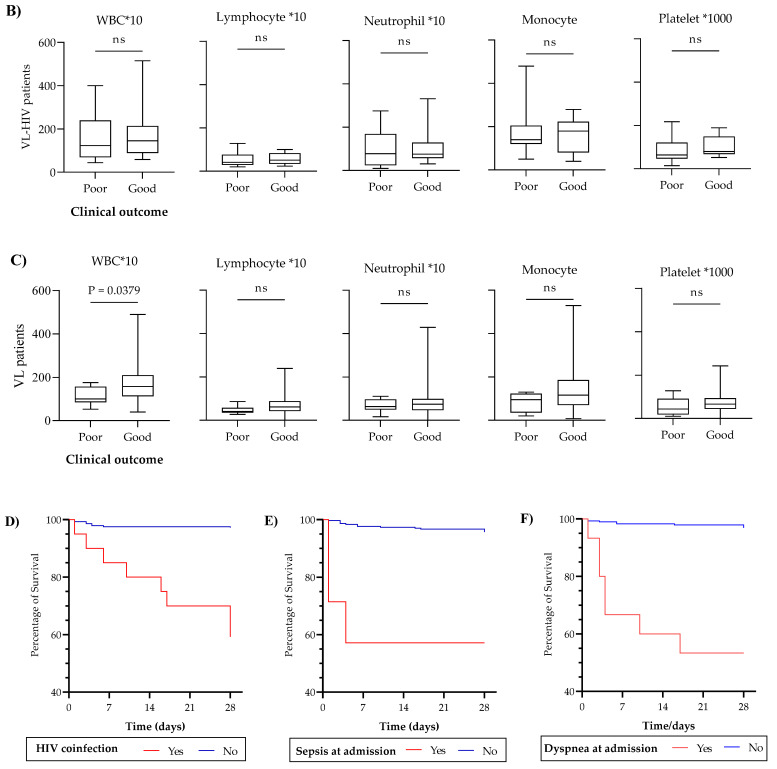
(**A**) End-of-treatment clinical outcomes, (**B**,**C**) leukocyte and platelet counts with respect to end-of-treatment clinical outcomes among VL patients with and without HIV coinfection. ns, statistically not significant. * 10, the presented cell count value should be multiplied by 10 to obtain the actual cell count. ns, not significant. (**D**–**F**) survival curves among VL patients with HIV, sepsis, and dyspnea, respectively.

On the other hand, a 17-day combination therapy with SSG and paromomycin (11 mg/kg/day) was given to 71.1% of HIV-negative VL cases (*n* = 2 deaths and 205 cured). The remaining HIV-negative VL cases were treated with a 30 mg/kg total dose of AmBisome monotherapy (*n* = 5 death and 14 cured), a 28-day course of SSG monotherapy (*n* = 1 death and 10 cured), and paromomycin and miltefosine combination therapy (this drug combination was used as part of a clinical trial study for 14 to 28 days duration) (*n* = 45 and all cases cured). Nine HIV-negative VL patients started SSG and paromomycin combination therapy, but the treatment was discontinued due to toxicity, and patients were further treated with AmBisome monotherapy (one treatment failure and eight cured). Overall, a total of 16 VL patients died during hospitalization, among which 8 (34.8%) were HIV coinfected VL patients and 8 (2.7%) were HIV-negative VL cases. At the time of discharge, 52.2% of HIV coinfected VL cases (*n* = 12) and 96.9% (*n* = 282) of HIV-negative VL patients were cured (Figure 4A).

HIV-negative VL patients with poor clinical outcomes had a significantly lower total leucocyte count as compared to those who were cured. However, the difference in this regard among HIV-positive patients was statistically insignificant (Figure 4B,C). Similarly, while the difference remains statistically non-significant (*p* = 0.312), HIV coinfected VL patients who were on ART for a short duration were more likely to have poor clinical outcomes as compared to those who were on ART for a long duration (190 ± 308 days vs. 1151 ± 1976 days) (Figure 3B). We then performed a bivariate analysis to determine independent predictors of poor clinical outcomes among all VL patients. Then, variables with significant associations in the bivariate analysis were included in the multivariate analysis. In this regard, despite the observed wide confidence interval, possibly due to the small number of the outcome variable, we observed sepsis (AOR, 17.2; 95% CI, 2.2 and 133.0), HIV (AOR, 30.2; 95%CI, 7.9 and 115.6), and dyspnea (AOR, 19.3; 95% CI, 3.9 and 96.2) at admission as independent predictors of poor clinical outcomes (Table 3). The probability of survival and death within the first month of therapy among VL patients with sepsis, HIV, and dyspnea at admission are also shown in Figure 4D–F.

## 4. Discussion

We conducted a retrospective analysis of laboratory characteristics, clinical features, and treatment outcomes of 314 patients who had been diagnosed and treated for VL at the University of Gondar LRTC. VL cases in this study were dominated by male patients (99.0% vs. 1.0%), possibly due to their frequent engagement in outdoor activities. Our finding for VL–HIV coinfection rate (7.0%) is comparable to reports from Tigray region, northwest Ethiopia (8.2%) [26], and Bahir, India (5.6%) [27]. However, the VL–HIV coinfection rate in this study is low compared to the VL–HIV coinfection estimated pooled prevalence (20.88% to 24.86%) from northwestern Ethiopia (reviewed in [13]) and 51% in Brazil [28]. On the other hand, as reviewed in [18], much lower VL–HIV coinfection rates than our finding have been reported in Kenya (1.4%), South Sudan (2.5%), and Sudan (1.3%). The decrease in the VL–HIV coinfection rate in our study compared to previous reports in northwest Ethiopia might be partly attributed to the war in northern Ethiopia and restrictions on internal movement during the COVID-19 pandemic, both of which might have affected the massive movement of seasonal mobile workers who have largely been linked to the high VL–HIV coinfection rate in the region [4,29,30]. In addition, the reported decreasing trends in the prevalence of HIV in Ethiopia may perhaps contribute to the decrease in the VL–HIV coinfection rate [14,31]. Any conclusions on this, however, should be interpreted with caution as there are no reliable data on the current national HIV prevalence in Ethiopia. Furthermore, HIV coinfection rates vary from place to place within a region, and the lack of reliable national data makes it challenging to show the trends on this.

Fever, which is one of the classical symptoms of VL, was less frequently observed in HIV coinfected VL cases. The findings are consistent with previous reports [19,28,32,33]. As stated elsewhere [28], the absence of fever in some HIV coinfected VL patients is likely due to the lack of proliferative response of mononuclear cells. Given that fever is one of the main markers for the initial diagnosis of VL, its absence in VL–HIV coinfected cases will pose diagnostic difficulties and delay the initiation of treatment.

Most VL–HIV coinfected patients in this study were treated with AmBisome and miltefosine combination therapy, which has been reported to be safe and effective with very low initial parasitological failure rates compared to AmBisome monotherapy [34]. The overall end-of-treatment cure rate among the VL–HIV coinfected group who started AmBisome and miltefosine combination therapy was 54.5% (12/22). Our finding is lower than previous reports in northwest Ethiopia [35,36] and India [37], where cure rates of AmBisome and miltefosine combination therapy among HIV coinfected cases were reported to be 67–83.8% and 96%, respectively. The lower end-of-treatment cure rate of AmBisome and miltefosine combination therapy in our case might partly be attributed to the long duration of illness at the time of diagnosis among HIV coinfected cases. Likewise, as shown by others [28,32,38,39], the high rate of relapse among HIV coinfected VL patients in our study (26.1%) would also contribute to the lower cure rate.

On the other hand, HIV-negative VL patients in this study were mostly treated with SSG and paromomycin combination therapy, which is in line with the current therapeutic recommendation for VL in Ethiopia [40]. SSG and paromomycin combination therapy was included as a first-line treatment for VL patients, replacing SSG monotherapy due to its comparable effectiveness and shorter treatment duration of 17 days [41,42,43]. In agreement with previous reports [42,44,45], we found a high end-of-treatment cure rate of SSG and paromomycin combination therapy (99.0%) among HIV-negative VL patients.

The overall mortality rate among all VL patients at the time of admission to the hospital was 5.1%, which is comparable to reports from Gondar, Ethiopia (4.8%) [46] and Brazil (7.0%) [47]. Of note, higher case fatality rates (11% to 18%) than our finding were reported in previous studies from Ethiopia [26,48,49], yet reports from South Sudan (2.8%) [50] and Uganda (3.7%) [51] showed lower case fatality rates. The variation in the mortality rates among studies might be partly attributed to the differences in HIV coinfection rates, duration of illness, presence of comorbidities, change in the treatment modalities, and the sample size. Indeed, we only recorded deaths that occurred during admission, and this may underestimate the overall mortality rate. A systematic review and meta-analysis by Gebreyohannes and colleagues showed that the end-of-treatment mortality rate due to VL is about 9.0%, which increases to 17.8% in follow-up studies [52].

As reported by others [28,32,53,54,55,56], we found that sepsis, HIV, and dyspnea are independent predictors of poor clinical outcomes (death and treatment failures) of VL. The high drug-related toxicity experienced by HIV coinfected VL patients might also contribute to the lower therapeutic success [22,38,39]. Furthermore, the underlying immunosuppression, as shown by the high prevalence of leucopenia in our case and others [57,58,59], may predispose VL patients to develop intercurrent infections, which leads to sepsis [60]. Sepsis has been described as one of the main causes of death in VL patients [61,62]. In line with this, Santos-Oliveira et al. described that the immunopathogenesis of VL is attributed to the breakdown of the immunological mucosal barrier that leads to the entry of bacterial LPS into the circulation, which then causes a cytokine storm and chronic immune hyperactivation [63].

This study is not without limitations. We recorded baseline clinical and hematological data of VL patients at admission, but post-treatment and follow-up data were not included. Furthermore, comparative analysis of hematological and clinical variables was not conducted among HIV-positive and HIV-negative VL patients because the two groups were not paired and had significant differences in the age, duration of illness, and treatment regimen. Interpretation of the treatment outcomes in the HIV coinfected patients is limited by the lack of available CD4 and viral load measurements to assess the level of immunosuppression and effectiveness of antiretroviral therapy.

## 5. Conclusions

HIV-negative VL patients treated with SSG and PM combination therapy showed good end-of-treatment responses. The low end-of-treatment responses among HIV coinfected VL patients is an alarming signal, and it suggests the need for alternative therapies to improve the clinical outcomes in this patient group. We believe that further studies are required with optimal sample size and paired samples to determine the actual treatment effects. Furthermore, special attention is required in the management of VL patients with sepsis, HIV, and dyspnea to improve patient prognosis.

## Figures and Tables

**Table 1:** Subject characteristics and clinical data among VL patients with and without HIV coinfection at the LRTC, Gondar, Ethiopia.

	VL–HIV, *n* = 23	VL, *n* = 291
Sex (male/female)	23:0	288:3
Age in years (mean ± SD)	35.30 ± 9.79	24.37 ± 7.16
Residence (rural/urban)	23:0	275:16
Ethnic group (Amhara/other)	23:0	290:1
Marital status (married/unmarried)	1:22	19:272
Duration of illness, days (mean ± SD)	62.26 ± 44.28	39.09 ± 29.83
Duration of illness >2 months (*n*, %)	9 (39.1%)	33 (11.3%)
Relapse (*n*, %)	6 (26.1%)	3 (1.0%)
PG in spleen (median, IQR)	5 (3)	2 (2)
Spleen PG ≥ 4 (*n*, %)	13 (72.2%)	58 (24.0%)
Spleen size (mean ± SD)	7.26 ± 3.25	8.50 ± 3.95
Liver size (mean ± SD)	3.50 ± 1.24	3.86 ± 1.65
Leucopenia (*n*, %)	20/23 (87.0%)	276/291 (94.8%)
Lymphocytopenia (*n*, %)	20/21 (95.2%)	225/273 (82.4%)
Neutropenia (*n*, %)	16/20 (80.0%)	246/268 (91.8%)
Monocytosis (*n*, %)	1/18 (5.6%)	0/218 (0.00%)
Anemia (*n*, %)	21 (91.3%)	247/289 (85.5%)
Thrombocytopenia (*n*, %)	17/22 (77.3%)	252/287 (87.8%)

**Table 2:** Clinical presentations, underlying medical conditions, and hematological data among VL patients with and without HIV coinfection at the time of admission at the LRTC, Gondar, Ethiopia.

	VL–HIV (*n*, %), *n* = 23	VL (*n*, %), *n* = 291
Clinical presentations		
Fever lasting more than 2 weeks	18 (78.3%)	286 (98.3%)
Abdominal pain	7 (30.4%)	85 (29.2%)
Abdominal swelling	10 (43.5%)	152 (52.2%)
Diarrhea	10 (43.5%)	49 (16.8%)
Cough	16 (69.6%)	166 (57.0%)
Jaundice	1 (4.3%)	50 (17.2%)
Pallor	3 (13.0%)	84 (28.9%)
Lymphadenopathy	3 (13.0%)	11 (3.8%)
Splenomegaly	23 (100.0%)	284 (97.6%)
Hepatomegaly	12 (52.2%)	119 (40.9%)
Weakness	1 (4.3%)	15 (5.2%)
Weight loss	16 (69.6%)	105 (36.1%)
Anorexia	17 (73.9%)	115 (39.5%)
Oedema	3 (13.0%)	78 (26.8%)
Joint pains	1 (4.3%)	10 (3.4%)
Dyspnea	5 (21.7%)	11 (3.8%)
Easily fatigable	8 (34.8%)	86 (29.6%)
Epistaxis	1 (4.3%)	16 (5.5%)
Vomiting	1 (4.3%)	10 (3.4%)
Malnutrition	22 (95.7%)	224/265 (84.5%)
Severe malnutrition	11/22 (50.0%)	67/224 (29.9%)
Underlying medical conditions		
Intestinal Parasite	6 (26.1%)	63 (21.6%)
Malaria		1 (4.3%)
Pneumonia	10 (43.5%)	52 (17.9%)
Tuberculosis	5 (21.7%)	11 (3.8%)
Sepsis	0 (0.0%)	7 (2.4%)
Urinary tract infections	0 (0.0%)	15 (5.2%)
COVID-19	1 (4.3%)	0 (0.0%)
Diabetes mellitus	0 (0.0%)	1 (0.3%)
Acute Kidney Injury	2 (8.7%)	2 (0.7%)
HBV infection	0 (0.0%)	3 (1.0%)
HCV infection	0 (0.0%)	5 (1.7%)
Acute gastro enteritis	1 (4.3%)	8 (2.7%)
Hyper-reactive malarial splenomegaly	0 (0.0%)	8 (2.7%)
Asthma	0 (0.0%)	1 (0.3%)
Hematological data		
WBC count (mean ± SD)	1745.65 ± 1215.67	1677.35 ± 814.57
Lymphocyte count (mean ± SD)	555.56 ± 293.43	705.31 ± 363.10
Neutrophil count (mean ± SD)	1044.32 ± 888.16	836.91 ± 493.23
Monocyte count (mean ± SD)	202.33 ± 168.48	140.23 ± 90.36
Mixed cell count (mean ± SD)	405.25 ± 134.00	186.95 ± 179.35
Hgb, g/dL (mean ±SD)	8.60 ± 2.20	8.69 ± 2.12
Platelet, count × 103/μL (mean ± SD)	96.36 ± 55.78	75.09 ± 45.83
BUN, mg/dL (mean ± SD)	24.28 ± 10.91	17.75 ± 14.55
AST, IU/L (mean ± SD)	63.30 ± 52.80	109.25 ± 104.69
ALT, IU/L (mean ± SD)	35.05 ± 27.31	60.30 ± 69.37
Na, mEq/L (mean ± SD)	134.18 ± 5.20	133.18 ± 7.97
K, mEq/L (mean ± SD)	3.68 ± 0.86	3.65 ± 0.61
Creatinine, mg/dL (mean ± SD)	1.20 ± 0.47	1.83 ± 7.26

SD, standard deviation; IU/L, international units per liter.

**Table 3:** Bivariate and multivariate analysis of predictors of poor clinical outcomes among VL patients during the time of admission at LRTC, Gondar, Ethiopia.

	Poor Outcome, *n* (%)	Good Outcome, *n* (%)	COR (95% CI)	AOR (95% CI)
Sepsis	3 (15.0%)	4/294 (1.4%)	13 (2.65, 61.79)	14 (1.55, 126.51)
HIV	11/20 (55.0%)	12/294 (4.1%)	29 (10.02, 82.36)	32 (8.45, 122.95)
Dyspnea	8/20 (40.0%)	8/294 (2.7%)	24 (7.64, 74.34)	18 (3.65, 87.40)
Hepatomegaly	13 (65.0%)	118 (40.1%)	3 (1.07, 7.15)	
Anorexia	14 (70.0%)	118 (40.1%)	3 (1.30, 9.31)	

COR, crude odds ratio; AOR, adjusted odds ratio; CI, confidence interval.

## Data Availability

Data is contained within the article.
